# A cross-sectional study of nausea in functional abdominal pain: relation to mucosal mast cells and psychological functioning

**DOI:** 10.1186/s12876-020-01291-2

**Published:** 2020-05-11

**Authors:** Craig Friesen, Meenal Singh, Vivekanand Singh, Jennifer V. Schurman

**Affiliations:** 1 Division of Gastroenterology, Hepatology, and Nutrition, Children’s Mercy Kansas City, 2401 Gillham Road, Kansas City, MO 64108 USA; 2 Department of Pathology and Laboratory Medicine, Children’s Mercy Kansas City, 2401 Gillham Road, Kansas City, Missouri USA; 3 Division of Developmental and Behavioral Sciences, Children’s Mercy Kansas City, 2401 Gillham Road, Kansas City, MO 64108 USA

**Keywords:** Functional dyspepsia, Irritable bowel syndrome, Mast cells, Anxiety, Somatization

## Abstract

**Background:**

Nausea is a common symptom in youth with chronic abdominal pain. The aims of the current study were to assess: 1) the frequency of nausea in patients with functional dyspepsia (FD) and irritable bowel syndrome (IBS), respectively, as defined by Rome IV criteria; and, 2) relationships between nausea and mucosal inflammation as defined by antral and duodenal eosinophil and mast cell densities. A secondary aim was to assess relationships between nausea and other gastrointestinal symptoms, non-gastrointestinal somatic symptoms, and psychological dysfunction.

**Methods:**

Records from patients with pain associated functional gastrointestinal disorders were retrospectively reviewed for gastrointestinal and somatic symptoms and anxiety, depression, and somatizations scores as assessed by the Behavior Assessment System for Children (BASC-2). In addition, previous gastric and mucosal biopsies were assessed for mast cell and eosinophil densities, respectively.

**Results:**

250 patients, ages 8 to 17 years, were assessed. Nausea was reported by 78% and was equally prevalent in those with FD alone, those with IBS alone, and those with both FD and IBS. Nausea was associated with increased mean (21.4 vs. 17.5) and peak (26.2 vs. 22.9) duodenal mast cell densities as compared those without nausea. Nausea was also associated with a wide variety of individual gastrointestinal symptoms, as well as headaches, fatigue, and dizziness. Lastly, nausea was associated with elevated self-report scores for anxiety (55.2 vs. 50.0), depression (50.2 vs. 46.1), and somatization (70.3 vs. 61.8).

**Conclusions:**

Nausea is common in children and adolescents with pain-associated FGIDs as defined by Rome IV and is not unique to either FD or IBS. Nausea is associated with increased mucosal mast cell density, non-gastrointestinal somatic symptoms, and psychologic dysfunction.

## Background

Chronic nausea is common in children, reported by 15.9% in a community sample [1]. For children who fulfill Rome III criteria for a pain-associated functional gastrointestinal disorder (FGID), the prevalence rate is even higher (36%) [1]. This makes sense, as nausea tends to be associated with other gastrointestinal symptoms, including worse abdominal pain [2]. In youth with functional abdominal pain, co-morbid nausea has been associated with more somatic complaints, greater psychological dysfunction, increased disability, and poorer prognosis, making this an important symptom to understand in its own right [2].

The observed relationships between abdominal pain and nausea would suggest at least some shared pathophysiology. One possible mechanism would be mucosal inflammation (e.g. mast cells, eosinophils) which has been implicated in functional dyspepsia (FD) and irritable bowel syndrome (IBS) but unstudied in relation to nausea. Mast cells have been extensively evaluated in adults with IBS. Results have demonstrated increased mast cell density in the colon, ileum, and duodenum with increased mast cell degranulation [3–6]. Further, the density of mast cells in close proximity to neurons correlates with pain frequency and intensity in adults with IBS [5]. Mast cells and eosinophils also have been implicated in FD [7]. A recent systematic review and meta-analysis of 37 studies concluded that FD is associated with increases in both mast cells and eosinophils in the antrum and duodenum [8]. Increased eosinophil and mast cell degranulation has been demonstrated in both adults and children with FD, further implicating these two cells in this condition [9–12].

Mast cells, in particular, would have the potential to increase nausea in patients with FD. Nausea is a common symptom in patients with mastocytosis or mast cell activation syndrome as mast cells release cytokines which can lead to nausea [13–15]. In patients with eosinophilic esophagitis, higher tryptase levels are associated with increased nausea frequency [16]. Furthermore, in youth with FD, increased mast cell density is associated with slower gastric emptying and increased gastric dysrhythmia [11]. Gastric dysrhythmia has been associated with nausea [17, 18].

Previous studies of nausea have assessed patients with FGIDs associated with abdominal pain as defined by Rome III criteria. The criteria have undergone some significant revisions in the most recent Rome IV criteria, particularly in how FD and IBS are defined. Previously, under Rome III, FD was defined as upper abdominal pain or discomfort unrelated to stools [19]. Under Rome IV, FD is defined by the presence of: 1) epigastric abdominal pain unrelated to stools; 2) early satiety; and/or, 3) postprandial fullness [20]. IBS is defined by the presence of pain related to a change in: 1) stool frequency; 2) stool consistency; or, 3) pain with stooling. Previously, Rome III criteria required two of these symptoms to be present, while Rome IV criteria only requires one of these symptoms to be present [19, 20]. Changes in criteria may be important in assessing associations between nausea and FGIDs. For example, Kovacic and colleagues evaluated children with abdominal pain and nausea to determine whether symptom profiles fulfilled Rome III FD criteria, and found that 87% met adult FD criteria while only 29% met pediatric criteria [21].. This is relevant because the Rome III adult FD criteria are akin to the Rome IV pediatric FD criteria. Thus, it is likely that the diagnosis rate and group composition of patients included in studies using Rome III versus Rome IV pediatric criteria is quite different and, consequently, earlier findings regarding the relationship between abdominal pain and nausea based on Rome III classification many not be generalizable moving forward.

The major aims of the current study were to assess: 1) the frequency of nausea in patients with FD and IBS, respectively, as defined by Rome IV criteria; and, 2) relationships between nausea and mucosal inflammation as defined by antral and duodenal eosinophil and mast cell densities. A secondary aim was to assess relationships between nausea and other gastrointestinal symptoms, non-gastrointestinal somatic symptoms, and psychological dysfunction. Understanding the relationship between nausea, the new Rome IV diagnostic criteria, and inflammation will be important in order to determine if Rome IV provides cleaner diagnostic categories as related to nausea and to evaluate whether nausea should be viewed as a treatment target separate from abdominal pain or if the two should be viewed as co-morbid conditions with shared inflammatory pathophysiology.

## Methods

The study retrospectively evaluated 250 consecutive patients diagnosed with an abdominal pain-associated FGID by a single board-certified pediatric gastroenterologist in an abdominal pain clinic at Children’s Mercy Kansas City. The study was approved by the Institutional Review Board Children’s Mercy Kansas City and has been performed in accordance with the ethical standards as laid down in the 1964 Declaration of Helsinki and its later amendments. Patients ranged in age from 8 to 17 years and all reported abdominal pain which occurred at least weekly for a minimum of 8 weeks. All patients completed a standard questionnaire as part of routine clinical evaluation. The questionnaire contained specific questions regarding symptoms required to classify patients according to Rome IV criteria, as well as other gastrointestinal symptoms and non-gastrointestinal somatic symptoms.

As part of routine clinical care, patients completed the Behavior Assessment System for Children – Second Edition (BASC-2) to assess for symptoms of anxiety, depression, and somatization [22]. The BASC-2 has demonstrated criterion-related and construct validity, has good internal consistency for most individual subscales, and is widely used in both clinical and research settings [22]. Standardized T scores for the self-report depression, anxiety, and somatization subscales were used for the current study. Self-report BASC data was available for 234 patients (94%).

A total of 208 patients (83%) had undergone an esophagogastroduodenoscopy with biopsies. All had failed to respond to acid reduction therapy. A minimum of 2 biopsies had been obtained from each of the antrum and the duodenum. All patients were negative for nodularity, erosions, ulcers, and Helicobacter pylori. Antral and duodenal biopsy specimens were assessed for eosinophil and mast cell densities, respectively. Eosinophils were assessed utilizing hematoxylin and eosin (H&E) stained slides obtained as part of routine care. An immunohistochemical stain for tryptase was utilized to determine mast cell densities. A mouse monoclonal antibody, anti-human mast cell tryptase, from Dako (clone AA1) was used at a dilution of 1:2000 on the Bond automated immunostainer following the routine immunohistochemistry protocol.

To determine eosinophil and mast cell densities, sections were initially scanned at a low magnification (10X) to determine areas of subjective maximal density. Using an Olympus CH30 microscope with a combination of 40x objective and 10x eyepiece (400X), lamina propria cells were counted in five consecutive high-power fields (hpf). Only eosinophils demonstrating a nucleus were enumerated. Mast cells of both spindled and epithelioid forms showing a nucleus were enumerated. Peak and mean cell densities were determined for the eosinophils and mast cells, respectively, in both the antrum and the duodenum. All cell counts were performed by a single observer (MS).

### Statistical analysis

SPSS version 23 (SPSS INc., Chicago, IL) was used to perform statistical analysis. The frequencies of a positive report of nausea were compared between patients meeting criteria for FD only, those meeting criteria for IBS only, and those meeting criteria for both FD and IBS (FD/IBS overlap) by chi square analysis. Mean and peak eosinophil and mast cell densities, as well as anxiety, depression, and somatization T scores, respectively, were compared for patients reporting nausea versus those who did not. For continuous variables, normality was assessed by utilizing Shapiro-Wilk. For parameters with normal distributions, significance was assessed by the student’s t test. For parameters with non-normal distributions, significance was assessed by Mann-Whitney U. In addition, effects sizes were determined utilizing cohen’s d for all parameters with differences determined to be statistically significant. Frequencies of specific gastrointestinal and non-gastrointestinal somatic symptoms also were compared between these groups by chi square analysis. A *p* value <.05 was considered statistically significant for all analyses.

## Results

A total of 250 consecutive patients presenting with chronic abdominal pain were evaluated. Patients ranged in age from 8 to 17 years (mean 13.2 ± 2.6 years) and 73% were female. Patient reported pain duration ranged from 2 to 190 months (mean 29.9 ± 38.0 months). The pain was reported as daily by 76%, several times per week by 17%, and weekly by 8%. Rome IV criteria for FD were fulfilled by 86%, IBS criteria were fulfilled by 58%, and both FD and IBS criteria were fulfilled by 50%. Frequencies for other gastrointestinal symptoms are shown in Table 1.

**Table 1:** Frequencies of specific gastrointestinal symptoms in patients with abdominal pain-associated functional gastrointestinal disorders (*N* = 250)

Symptom	% of Patients
Pain with eating	73
Early satiety	70
Waking at night with pain	56
Postprandial bloating	51
Heartburn	38
Vomiting	26
Diarrhea	22
Constipation	15

Nausea was reported by 78% of patients. Nausea was seen in similar proportions of FD and IBS patients being reported by 81% of patients fulfilling FD criteria only, 75% of patients fulfilling IBS criteria, and 80% of patients fulfilling both FD and IBS criteria (*p* = 0.814). Of those patients reporting nausea, it was daily in 37%, several times per week in 37%, weekly in 9%, and less than weekly in 17%. Nausea increased with meals on at least some occasions in 27.7% of patients. Vomiting was reported by 33.6% of patients.

Antral and duodenal mucosal specimens were available for 208 patients. For ease of interpretation, we present mean and peak duodenal mast cell densities, *p* values for t tests, and standard error of differences for patients with and without nausea in Table 2. Nausea was associated with increased duodenal mast cell density. There were no group differences for antral mast cell densities or eosinophil densities in either the antrum or duodenum. Antral mean and peak mast cell densities were normally distributed while all other densities were not. Non-parametric methods confirmed significance for both mean (*p* = 0.034) and peak (*p* = 0.029) duodenal mast cell densities. Effect sizes were small to medium for both mean (cohen’s d = 0.303) and peak (cohen’s d = 0.383) duodenal mast cell densities.

**Table 2:** Mucosal eosinophil and mast cell densities in patients with and without nausea

Variable	Nausea positive (*N* = 169)	Nausea negative (*N* = 39)	*p* value
Mean antral eosinophils	10.5 ± 9.2	10.0 ± 7.0	NS
Peak antral eosinophils	15.0 ± 12.0	15.1 ± 9.6	NS
Mean duodenal eosinophils	21.1 ± 11.9	20.9 ± 8.7	NS
Peak duodenal eosinophils	28.1 ± 14.7	28.2 ± 12.0	NS
Mean antral mast cells	15.6 ± 5.6	15.1 ± 6.1	NS
Peak antral mast cells	20.2 ± 7.3	19.9 ± 8.2	NS
Mean duodenal mast cells	21.4 ± 17.1	17.5 ± 6.3	.02
Peak duodenal mast cells	26.2 ± 9.7	22.9 ± 7.4	.02

The frequencies for other gastrointestinal and non-gastrointestinal somatic symptoms in patients with and without nausea are shown in Table 3. Mean self-report scores, standard error of differences, and effect sizes for anxiety, depression and somatization in patients with and without nausea are shown in Table 4, along with *p* values for t tests. All 3 of these subscales were elevated in patients with nausea relative to those patients without nausea. Somatization scores were normally distributed while those for anxiety and depression were not. Non-parametric methods confirmed significance for both anxiety (*p* = 0.004) and depression (*p* = 0.001) scores. Effect sizes were medium for anxiety (cohen’s d = 0.485) and depression (cohen’s d = 0.461) and large for somatization (cohen’s d = 0.821).

**Table 3:** Frequencies of gastrointestinal and non-GI somatic symptoms in patients with and without nausea

Symptom	Nausea positive (*N* = 195)	Nausea negative (*N* = 55)	*p* value
Pain with eating	73.2	73.1	NS
Early satiety	73.1	57.6	.03
Postprandial bloating	56.6	30.8	.001
Vomiting	32.8	0	<.001
Heartburn	42.9	17.3	.001
Change in stool frequency	23.2	5.8	.003
Change in stool form	30.3	15.4	.03
Pain relief with stool	40.4	48.1	NS
Headache	74.3	52.0	.003
Fatigue	56.3	34.0	.006
Dizziness	60.0	21.7	<.001

**Table 4:** Self-report BASC subscales in patients with and without nausea

Variable	Nausea positive (*N* = 184)	Nausea negative (*N* = 50)	*P* value
Anxiety	55.2 ± 11.4	50.0 ± 10.0	.002
Depression	50.2 ± 9.7	46.1 ± 8.0	.006
Somatization	70.3 ± 10.6	61.8 ± 10.1	<.001

## Discussion

Nausea is a very common symptom in children and adolescents fulfilling Rome IV criteria for FD and/or IBS, respectively. Nausea was equally common in the two conditions being present in approximately 80% of each in the current study. The frequency is similar to the 87% previously reported in youth fulfilling Rome III adult FD criteria, which are akin the Rome IV pediatric FD criteria [21]. Nausea was associated with a number of specific gastrointestinal symptoms. These include dyspeptic (early satiety and postprandial bloating) and IBS (change in stool form and frequency) symptoms, as well as symptoms not specific to either condition (vomiting and heartburn). This further demonstrates that nausea is not unique to any specific abdominal pain-associated FGID.

In the current study, we have demonstrated an increase in duodenal mast cells in FD patients with nausea, beyond what would have presumably occurred with the abdominal pain associated functional gastrointestinal diagnosis alone. Whether the observed differences are clinically significant is not clear. This association with nausea appears unique to mast cells, as eosinophil density did not differ between those with and without nausea in the current study. This indicates that relationships between inflammatory cells and nausea are unique and separate from FGID more generally, as previous studies in children and adults with FD have demonstrated increased duodenal eosinophil density [8]. While we are only able to assess an association in the current study, it is possible that mast cell activation could induce nausea by direct or indirect effects of released mediators on central nervous system functioning or by altering gastric motor function. We have previously found an association between increased mucosal mast cell density and gastric dysrhythmia and slower gastric emptying [11]. Jericho and colleagues have reported an association between nausea and slow gastric emptying in children and adolescents [23]. Likewise, nausea has been associated with gastric dysrhythmia [17, 18]. As nausea can be a distressing symptom, it is also possible that nausea induced a physical stress response with subsequent mast cell recruitment. Alternatively, the autonomic nervous system may play a role. Tarbell and colleagues previously reported an association between chronic nausea and autonomic dysfunction [24]. Altered autonomic function has been reported in children with both IBS and FD [25, 26].

In the current study, nausea was associated with higher self-reported scores for anxiety, depression, and somatization. Previous studies also have found an association for nausea (alone or in combination with abdominal pain) with anxiety, depression, and somatization [2, 24, 27]. In addition, nausea has been associated with poor school and social functioning [21]. In the current study, nausea also was associated with increased frequencies of specific non-gastrointestinal somatic complaints, including headache, fatigue, and dizziness. Associations with dizziness and fatigue have previously been reported with nausea [27]. The association of nausea with these somatic symptoms suggests a common pathophysiologic process. The possibility of mast cell activation was raised above. We have previously demonstrated an association between mast cell density and somatization as well as anxiety and depression in youth with FD [28]. Anxiety and depression have been associated with mast cell density and degranulation in adults with FD [9]. Another proposed mechanism is autonomic dysfunction, which has been associated with anxiety in children with nausea and those with abdominal pain [24, 25].

A strength of the current study is the systematic collection of symptoms and the availability of mucosal specimens for analysis in a large population of patients with abdominal pain. A limitation of the current study is that 17% of the patients did not have biopsies to be evaluated. This could have introduced some possible bias, as endoscopy is not routinely performed in patients with IBS alone and, thus, biopsies in the locations of interest were more likely to have been obtained in patients with FD (with or without IBS). As nausea was equally prevalent in FD and IBS in our patient group, this oversampling of patients with FD is unlikely to have had a significant impact on our results and, at a minimum, the current study would at least provide information on nausea’s association with mucosal inflammation relevant to the large group of patients with FD. Another limitation of the current study is that cell counts represent only a gross measure of inflammation; as such, they do not allow an assessment of general activation or an assessment of specific mediator concentrations to determine which mediators, if any, are contributing to the symptom of nausea. Understanding specific mediator contributions might indicate specific therapeutic targets. It should be emphasized that this is an observational, cross-sectional study and therefore, cause-and-effect cannot be imputed. Lastly, as validated pediatric measures of nausea severity were not available at the time of data collection, we were unable to assess relationships for varying degrees of nausea severity. A measure is now available and should be incorporated into future studies [29].

## Conclusions

Nausea is common in children and adolescents with pain-associated FGIDs as defined by Rome IV and is not unique to either FD or IBS. Nausea is associated with increased mucosal mast cell density, non-gastrointestinal somatic symptoms, and psychologic dysfunction. Because of the distressing nature of nausea, and its negative associations with school and social functioning, it represents a treatment target of importance in its own right. Prospective studies are needed to determine what is driving the association between nausea, mast cells, and psychological dysfunction, specifically to assess whether there is a cause-and-effect relationship. Future studies should evaluate specific mast cell mediators in relation to nausea and evaluate the efficacy of medications directed at mast cell stabilization or antagonism of specific mast cell mediators in the treatment of nausea.

## Data Availability

Data used by or generated in this study will be available upon request to the corresponding author.

## Abbreviations

FGID: Functional gastrointestinal disorder
FD: Functional dyspepsia
IBS: Irritable bowel syndrome
BASC: Behavior Assessment System for Children
